# Outcome Patterns of SPT-SAFE and DBT-BI in Adolescents with Suicidal Ideation and Non-Suicidal Self-Injury: A Retrospective School-Based Study

**DOI:** 10.3390/bs16060916

**Published:** 2026-06-03

**Authors:** Hyeonjeong Kwak, Unkyoung Ahn

**Affiliations:** 1 Department of Psychology, Dankook University, Cheonan 31116, Republic of Korea; kwakhj@dankook.ac.kr; 2 Institute for Mental-Health, Dankook University, 119 Dandae-ro, Cheonan 31116, Republic of Korea

**Keywords:** adolescent mental health, non-suicidal self-injury, suicidal ideation, sandplay therapy, dialectical behavior therapy, school-based intervention, impulsivity

## Abstract

Background/Objectives: Adolescent suicidal ideation and non-suicidal self-injury (NSSI) are major public health concerns, yet differences between active interventions are often modest. This retrospective study provides preliminary descriptive evidence regarding short-term symptom change across two school-based intervention pathways—Sandplay Therapy with Suicidal ideation and Self-injury Focused Engagement (SPT-SAFE) and a Dialectical Behavior Therapy-informed Brief Intervention (DBT-BI)—in a clinical sample of middle- and high-school adolescents with suicidal ideation and NSSI. Methods: Archival clinical records from 112 adolescents treated in a school-based suicide prevention center were analyzed retrospectively. The sample included 52 adolescents who received SPT-SAFE and 60 who received DBT-BI. Outcomes included suicidal ideation, NSSI frequency, depressive symptoms, anxiety, aggression, impulsiveness, and self-concept. Pre–post changes were examined using mixed-design ANOVAs, with baseline-adjusted ANCOVAs conducted as supplementary analyses. Given the retrospective and clinically assigned design, findings were interpreted as observational associations rather than causal treatment effects. Results: Both intervention groups showed significant pre–post improvements across multiple outcome domains. Overall between-group differences were limited. A nominal, uncorrected Group × Time interaction was observed for impulsiveness, with the DBT-BI group showing a descriptively larger pre–post decrease than the SPT-SAFE group. However, this effect was small and did not remain statistically significant after Bonferroni correction or Benjamini–Hochberg false discovery rate correction across the seven interaction tests. No other outcome showed a multiplicity-adjusted Group × Time interaction. Conclusions: The present study did not provide robust multiplicity-adjusted evidence for differential treatment effects between SPT-SAFE and DBT-BI. The nominal impulsivity finding should be interpreted only as a small, uncorrected exploratory signal for future hypothesis generation. The contribution of this study is therefore descriptive and preliminary, characterizing short-term symptom change in a treatment-engaged adolescent completer sample within a routine school-based service system, rather than supporting domain-specific comparative efficacy or domain-sensitive intervention planning.

## 1. Introduction

### 1.1. Background

Adolescent suicidal ideation and non-suicidal self-injury (NSSI) are major public health concerns because of their increasing prevalence and their strong association with subsequent suicide attempts and mortality (Nock et al., 2013; Ribeiro et al., 2016). These problems are especially salient during adolescence, a developmental period marked by heightened emotional reactivity, ongoing maturation of cognitive control systems, and increasing demands for self-regulation (Casey et al., 2008; Steinberg, 2005). In clinically severe adolescent samples, this developmental imbalance may make vulnerabilities such as impulsivity, emotion dysregulation, and behavioral dysregulation particularly important targets for intervention.

School-based mental health services occupy a distinctive position in youth suicide prevention. Unlike specialty outpatient clinics, school-based services often function as early-access systems in which students are identified through school referral pathways, assessed for immediate safety concerns, and connected to brief interventions under conditions of limited time, high clinical urgency, and ongoing coordination with caregivers and school personnel (Fazel et al., 2014). Under these conditions, school-based clinicians must often respond pragmatically to multiple areas of risk and functioning within limited timeframes and available service resources.

Over the past several decades, a wide range of psychosocial interventions has been developed for suicidal ideation and self-injurious behaviors. However, meta-analytic evidence indicates that overall treatment effects are generally modest, and no single intervention has shown consistent superiority across studies (Fox et al., 2020; Wright-Hughes et al., 2025). Even in randomized trials and systematic reviews involving adolescents, differences between active treatment conditions are often small, time-limited, or statistically non-significant (Diamond et al., 2019; Mehlum et al., 2016; Ougrin et al., 2015). Taken together, this pattern supports the need for cautious descriptive studies that examine short-term changes across multiple clinical outcomes in heterogeneous school-based samples.

### 1.2. Limitations of Existing Intervention Research

Limited between-treatment differences in adolescent self-harm intervention studies may partly reflect the fact that active interventions often share core therapeutic elements, including therapeutic engagement, emotion regulation, problem-solving, and safety planning. This overlap is especially relevant in school-based and routine-care settings, where structured risk assessment, safety monitoring, and caregiver/school coordination are frequently embedded across intervention conditions. Accordingly, studies using active comparison groups may be less likely to detect broad superiority effects, even when clinically meaningful change occurs within each condition (Fox et al., 2020; Ougrin et al., 2015; Wright-Hughes et al., 2025).

### 1.3. Developmental and Severity-Related Context for School-Based Intervention

Developmental stage and clinical severity are relevant considerations when describing adolescents who present to school-based services with suicidal ideation and NSSI. Among adolescents with more recurrent NSSI and more severe suicidal ideation, behavioral dysregulation, impulsivity, and emotion dysregulation are commonly co-occurring clinical features, and clinicians in school-based settings must respond within limited timeframes while maintaining safety monitoring and caregiver–school coordination (Fazel et al., 2014). This context provides the background for the present study of adolescents who received SPT-SAFE or DBT-BI in a school-based service system.

### 1.4. Study Rationale and Objectives

Building on our related retrospective observational study of elementary-school students (Kwak & Ahn, 2026), the present study examined a distinct, non-overlapping middle- and high-school adolescent sample treated within the same school-based service system. This separation was intended to describe the adolescent cohort within its own clinical and service context rather than to test developmental differences between cohorts.

For descriptive clarity, the seven outcomes were grouped into four clinically defined areas: self-harm-related outcomes (suicidal ideation and NSSI frequency), emotional distress (depressive symptoms and trait anxiety), behavioral dysregulation (aggression and impulsivity), and self-related functioning (self-concept). This grouping was used only to organize the presentation of outcomes and was not intended to imply statistically distinct domains, treatment-specific mechanisms, or differential treatment effects.

SPT-SAFE is a school-based adaptation of nondirective sandplay therapy, whereas DBT-BI is a brief DBT-informed individual intervention. Both pathways incorporated shared safety-oriented procedures, including suicide-risk monitoring, safety planning, and caregiver/school coordination.

Accordingly, the present study was conducted as a retrospective descriptive analysis of short-term symptom change among treatment-engaged adolescent completers who received one of two school-based intervention pathways, SPT-SAFE or DBT-BI. The aims were to describe pre–post changes across multiple clinical outcomes and to report any observed uncorrected between-group patterns, interpreted only as exploratory findings.

## 2. Materials and Methods

### 2.1. Study Design and Relation to the Prior Report

The present study employed a retrospective observational comparative design based on archival clinical records derived from routine care within a school-based mental health service system. The study did not involve prospective recruitment, random assignment, or protocol-driven intervention delivery. All data were generated as part of standard clinical practice before the present analyses were conceived.

This analysis was conducted within the same clinical service context, ethics framework, and general study period as our related elementary-school retrospective observational study (Kwak & Ahn, 2026), but was restricted to a distinct middle- and high-school sample. The two studies therefore involved developmentally different populations drawn from separate analytic samples, and no participants included in the elementary-school study were included in the present analysis.

Although a pooled analysis across the elementary-school and adolescent cohorts was considered, the two cohorts were analyzed separately because they represented developmentally and clinically distinct populations. The elementary-school cohort and the present middle- and high-school cohort differed in developmental stage, clinical severity, and the likely salience of self-related versus behavioral-regulation domains. Pooling the samples could have obscured these developmentally meaningful clinical differences and produced an overall estimate that was less informative for school-based intervention planning. Therefore, the present study was designed as a separate cohort analysis focused on the adolescent sample, while using the prior elementary-school study as a related developmental and service-context reference point.

Accordingly, the present analyses were intended to characterize patterns of change across two clinically implemented intervention tracks in a naturalistic setting and to examine whether intervention-associated differences were more evident in specific outcome domains rather than at the global level. Given the retrospective and clinically assigned nature of the study, the findings are interpreted as observational associations within a real-world clinical context. No causal inferences regarding treatment effectiveness, comparative superiority, or differential efficacy between interventions are intended.

### 2.2. Setting and Participants

Clinical records were obtained from a school-based suicide prevention center (KASS), commissioned by the Chungcheongnam-do Office of Education, Republic of Korea. The center provides crisis-oriented assessment and brief intervention for students referred through school-based pathways because of suicidal ideation, NSSI, and related emotional or behavioral concerns.

Chungcheongnam-do is a large provincial education district comprising urban, suburban, and rural school communities. The center functioned as a regional referral and intervention hub, receiving students identified by schools as being at risk for suicidal ideation, NSSI, or related difficulties. During the study period, the service system covered middle- and high-school students referred from schools across the province, thereby providing a naturalistic context for examining intervention patterns under routine school-based care conditions.

For the present study, archival clinical records were reviewed for middle- and high-school students who received services at the center between 2022 and 2024.

Cases were considered eligible for inclusion if records documented the following: (1) enrollment in middle or high school; (2) sufficient literacy to complete Korean self-report measures; (3) documented NSSI within the six months preceding intake; (4) suicidal ideation without acute intent or an imminent suicide plan at intake; and (5) participation in at least six therapeutic sessions with both pre- and post-intervention assessment data available. These criteria were applied to identify cases in which clinically meaningful engagement with the intervention had occurred and outcome data were available for analysis.

Cases were excluded if records indicated: (1) acute suicidal intent requiring emergency or inpatient care; (2) a recent medically serious suicide attempt; (3) severe psychiatric instability requiring immediate intensive treatment; (4) absence of documented NSSI; (5) discontinuation of treatment prior to completion of the minimum session threshold; or (6) transfer to a higher level of care during the treatment episode.

**Figure 1:**
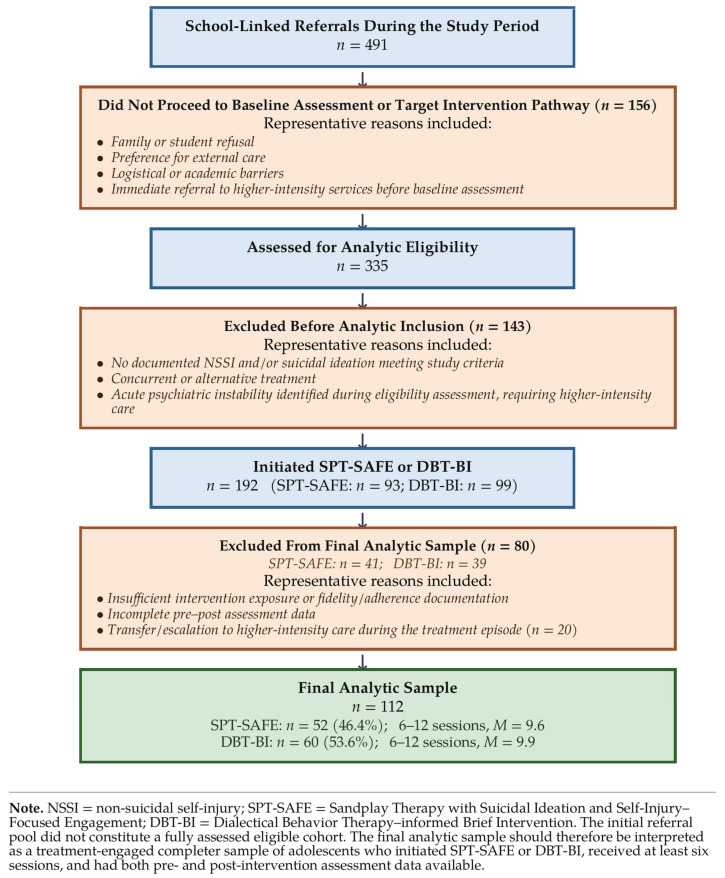
Flow diagram of participant selection and analytic inclusion.

After application of these criteria, the final analytic sample consisted of 112 adolescents, including 52 in the SPT-SAFE group and 60 in the DBT-BI group. The participant flow and analytic inclusion process are presented in Figure 1.

The final analytic sample represents a treatment-engaged completer sample rather than a representative sample of all 491 school-linked referrals. The reduction from 491 referrals to 112 analytic cases reflects multiple sequential stages: 156 referrals did not proceed to baseline assessment or the target intervention pathway; 143 of the remaining 335 assessed cases were excluded prior to analytic inclusion; 192 adolescents initiated SPT-SAFE or DBT-BI; and 80 of these initiators were excluded from the final analytic sample, including 20 who were transferred or escalated to higher-intensity care during the treatment episode. A full comparison of included versus excluded cases was not feasible because structured demographic, clinical, and outcome data were not consistently available across the excluded referral stages, particularly for referrals that did not proceed to baseline assessment or the target intervention pathway. The complete participant flow is presented in Figure 1, and safety-related events and higher-intensity care escalation are summarized in Supplementary Table S3.

### 2.3. Clinical Assignment and Decision-Making

Clinical assignment was not randomized but was determined as part of routine clinical decision-making within the school-based service system. Assignment occurred before outcome evaluation and independently of the present analyses. All participants underwent a standardized intake process, including structured suicide risk assessment, clinical interviews, and caregiver consultation, through which relevant clinical and contextual information was documented.

Assignment to SPT-SAFE or DBT-BI was based on multiple clinically relevant considerations rather than a single predefined criterion. These considerations included the severity and pattern of suicidal ideation and self-injury, behavioral dysregulation and impulsivity, need for structured behavioral stabilization, capacity for verbal versus symbolic expression, developmental and communication characteristics, prior treatment history, and comorbid emotional or behavioral difficulties. Contextual factors, including caregiver preference, school coordination needs, therapist availability, and service-level considerations, were also taken into account.

Adolescents presenting with acute suicide risk requiring immediate stabilization, such as imminent suicidal intent, medically serious suicide attempts, or need for emergency intervention, were not allocated to either intervention track and were referred to higher-intensity services according to standard clinical protocols. Accordingly, the present sample reflects adolescents considered clinically appropriate for outpatient-level structured intervention following initial risk screening and triage.

Baseline demographic and clinical characteristics relevant to treatment assignment are summarized in Table 1. These variables were examined to describe baseline comparability between groups, not to establish exchangeability. Because treatment assignment was embedded in routine clinical judgment, comparability on measured variables does not eliminate the possibility of clinically meaningful differences in unmeasured or partially documented factors. In particular, factors such as behavioral dysregulation, impulsivity, expressive style, or family and school context may have influenced both clinical assignment and subsequent treatment response.

Because these same clinical features may also predict subsequent change in impulsivity and behavioral regulation, the assignment process introduces a specific risk of confounding by indication. In particular, adolescents assigned to DBT-BI may have been those whom clinicians perceived as requiring more structured behavioral stabilization because of greater behavioral dysregulation, crisis instability, or readiness for skills-based work. Therefore, baseline comparisons were used only to describe measured group differences at intake and were not interpreted as demonstrating exchangeability between the intervention groups.

Although efforts were made within the service system to maintain a clinically balanced distribution of cases across intervention tracks, the non-randomized nature of assignment introduces the possibility of residual confounding, including confounding by indication. Accordingly, observed between-group differences are interpreted as observational associations within a naturalistic clinical context rather than as evidence of causal effects, comparative treatment superiority, or differential efficacy between interventions.

### 2.4. Interventions

Both interventions were delivered individually in weekly sessions of approximately 45–50 min. In the final analytic sample, participants received 6–12 sessions, with a mean dose of approximately 9–10 sessions in each intervention pathway.

#### 2.4.1. Shared Safety Procedures

Before intervention-specific treatment, all adolescents received standardized safety-oriented procedures as part of routine school-based suicide risk management. These procedures included structured suicide/NSSI risk assessment, individualized safety planning, session-by-session monitoring of suicidal ideation and NSSI-related risk indicators, caregiver/school coordination when clinically indicated, and referral or escalation to psychiatric or emergency services when acute risk was identified. These safety procedures were implemented across both intervention tracks and were not specific to either SPT-SAFE or DBT-BI.

#### 2.4.2. SPT-SAFE

SPT-SAFE was delivered as a school-based adaptation of nondirective sandplay therapy that integrated ongoing clinical attention to suicidal ideation and NSSI risk. This format reflected the routine school-based crisis-oriented service context, in which adolescents required both a protected symbolic-expressive space and continuous safety monitoring. Sessions generally included: (1) suicide/NSSI risk monitoring and emotional check-in; (2) tactile grounding or session introduction through contact with sand; (3) construction of a sand scene using miniature figures; (4) reflective observation of the sand scene; (5) optional verbal sharing or meaning exploration guided by the adolescent’s readiness; (6) review of emotional state and safety status; and (7) documentation of session content, safety status, and clinically indicated follow-up actions.

Therapists maintained core nondirective sandplay principles, including an empathic and nonjudgmental witnessing stance, respect for symbolic expression, and avoidance of imposed interpretation. Adolescents were not required to verbalize internal thoughts, painful emotions, shame-related experiences, suicidal content, or the personal meaning of symbolic scenes unless they chose to do so. However, therapists maintained explicit safety monitoring throughout the intervention and shifted to safety-oriented clinical procedures, including risk assessment, stabilization when needed, caregiver/school coordination, and referral or escalation to higher-intensity services when acute risk indicators emerged. Accordingly, SPT-SAFE should be understood as a safety-integrated, school-based adaptation of nondirective sandplay therapy, not as sandplay therapy delivered without suicide/NSSI risk management.

#### 2.4.3. DBT-BI

DBT-BI was delivered as a brief, school-based, DBT-informed individual intervention rather than as the full multicomponent DBT-A package. This abbreviated format reflected the routine school-based service context, in which adolescents were referred for crisis-oriented assessment and short-term intervention under constraints of limited time, caregiver–school coordination, and ongoing safety monitoring. Sessions therefore focused on DBT-informed individual-session procedures most directly relevant to immediate risk stabilization: (1) brief mindfulness-based stabilization; (2) review of suicidal ideation and NSSI using diary-card or monitoring information; (3) prioritization of target risk behaviors; (4) DBT-based chain analysis of recent risk episodes; and (5) collaborative practice of selected skills targeting identified risk behaviors. Skills practice was collaboratively selected according to the adolescent’s target risk behaviors and immediate stabilization needs, drawing as appropriate on emotion regulation, distress tolerance, problem solving, and impulse-control strategies. DBT-BI did not include weekly multifamily skills training groups, between-session telephone coaching, a formal DBT therapist consultation team, or a long-term comprehensive DBT-A treatment structure. Accordingly, DBT-BI should be understood as a DBT-informed brief intervention adapted to a school-based crisis-oriented service context, not as a test of comprehensive DBT-A.

#### 2.4.4. Therapist Teams, Supervision, and Fidelity/Adherence Monitoring

To reduce treatment contamination, SPT-SAFE and DBT-BI were delivered by separate therapist teams with no provider overlap. Each intervention was delivered by five licensed clinical or counseling psychologists. Across the final analytic sample, this corresponded to an average cumulative load of approximately 10–12 participants per therapist within each intervention track during the study period; this should not be interpreted as a concurrent caseload estimate. Model-specific supervision was provided through regular case consultation meetings focused on adherence to core intervention principles, safety monitoring, and clinical risk management. Manual-derived fidelity/adherence checklists for SPT-SAFE and DBT-BI are provided in Supplementary Tables S1 and S2, and approximately 20% of sessions were independently reviewed for adherence. However, because this was a retrospective clinical record study, session-level fidelity ratings were not systematically compiled into a quantitative database. Accordingly, aggregate fidelity scores cannot be reported and therapist-level effects cannot be formally modeled.

### 2.5. Outcome Measures

The assessment battery and scoring procedures followed the same general framework as our related elementary-school retrospective observational study (Kwak & Ahn, 2026). For all measures, higher scores indicated greater levels of the corresponding construct unless otherwise stated. For interpretive clarity, the seven outcome measures were grouped into four clinically defined domains prior to analysis: self-harm-related outcomes (SIQ-JR, FASM), emotional distress (CES-DC, STAI-T), behavioral dysregulation (AQ, BIS), and self-related functioning (PHCSCS). This grouping was based on the clinical function represented by each measure and on the theoretical relevance of these domains to school-based intervention planning for adolescents with suicidal ideation and NSSI.

The FASM was used as a dimensional self-injury frequency measure, not as a DSM-based diagnostic instrument for NSSI disorder. In this retrospective clinical record review, NSSI was defined as self-injurious behavior documented as occurring without suicidal intent. Inclusion in the analytic sample was determined retrospectively from intake clinical records, clinical interviews, documented risk assessments, and FASM suicidal intent follow-up item responses, rather than from FASM frequency responses alone. Cases with documented suicidal intent, imminent suicidal intent, or medically serious suicide attempts were referred to higher-intensity services in routine care and were not retained in the analytic sample. Because some behaviors included in the original FASM may require clinical contextualization to distinguish self-injurious acts from socially sanctioned body modification, FASM scores were interpreted as a dimensional self-injury frequency index rather than as a DSM-based diagnosis.

#### 2.5.1. Primary Outcomes

Suicidal ideation. Suicidal ideation was assessed using the Suicidal Ideation Questionnaire–Junior (SIQ-JR; Reynolds, 1988), a 15-item self-report measure rated on a 7-point scale. Higher scores indicate greater suicidal ideation. A Korean adolescent version has been reported (Y. S. Lee et al., 2004).

NSSI. NSSI was assessed using the Functional Assessment of Self-Mutilation (FASM; Lloyd, 1997), a self-report measure of self-injurious behaviors and their associated functions. A Korean version was used in the present study (Kwon & Kwon, 2017). For the present analyses, an overall NSSI frequency index was computed by summing method-specific frequency ratings, with higher scores indicating greater NSSI frequency.

#### 2.5.2. Secondary Outcomes

Depressive symptoms. Depressive symptoms were assessed using a Korean-language version of the 20-item CES-DC, adapted from the original CES-D for children and adolescents (Radloff, 1977; Faulstich et al., 1986). Higher scores indicate greater depressive symptoms.

Trait anxiety. Trait anxiety was assessed using a Korean-language version of the 20-item Trait Anxiety scale of the State-Trait Anxiety Inventory for Children (STAI-T; Spielberger et al., 1973). Items are rated on a 3-point scale, with higher scores indicating greater trait anxiety.

Aggression. Aggression was assessed using the Aggression Questionnaire (AQ; Buss & Perry, 1992). The Korean version consists of 27 items rated on a 5-point scale, with higher scores indicating greater aggression. Acceptable internal consistency has been reported in Korean samples (Seo & Kwon, 2002).

Impulsivity. Impulsivity was assessed using a 23-item shortened Korean version of the Barratt Impulsiveness Scale-11 (BIS-11; Patton et al., 1995). Items are rated on a 4-point scale, yielding a total score range of 23–92, with higher scores indicating greater impulsivity. The Korean BIS-11 has demonstrated acceptable reliability and validity in prior Korean samples (S. R. Lee et al., 2012).

Self-concept. Self-concept was assessed using a modified PHCSCS-based self-concept index consisting of 30 items. Item content was retained from the Piers–Harris self-concept item pool (Piers & Herzberg, 2002), drawn from Korean-translated PHCSCS/PHCSCS-2 materials informed by prior Korean adaptation work (Choi, 2013). The 30-item short-form structure was informed by Veiga and Leite (2016). The response format was changed from the original dichotomous format to a 6-point Likert scale. In the present sample, internal consistency was good (Cronbach’s α = 0.88). Scores were computed by summing item responses; higher scores indicate a more positive self-concept. Because this exact format has not been independently validated, self-concept findings are interpreted only as exploratory secondary outcome data and are not compared with PHCSCS-2 norms or cutoffs.

### 2.6. Statistical Analysis

All analyses were conducted using IBM SPSS Statistics, version 25. Baseline differences between the two intervention groups were examined descriptively using independent-samples *t*-tests; Welch’s *t*-test was applied when the homogeneity of variance assumption was not met. Because intervention assignment was not randomized, these baseline comparisons were not treated as evidence of exchangeability between groups, but rather as descriptive indicators of the magnitude of differences on measured variables at intake.

The primary longitudinal analyses used 2 × 2 mixed-design analyses of variance (ANOVAs) for each outcome, with Group (SPT-SAFE vs. DBT-BI) as the between-subjects factor and Time (pre- vs. post-intervention) as the within-subjects factor. The effect of principal interest was the Group × Time interaction, which was used to examine whether patterns of pre–post change differed between intervention groups across outcome domains.

To complement these analyses, one-way analyses of covariance (ANCOVAs) were conducted for each post-intervention outcome, with group entered as the fixed factor and the corresponding baseline score entered as a covariate. These ANCOVAs were intended as supplementary baseline-adjusted analyses rather than as the primary inferential framework. Accordingly, if the two analytic approaches diverged, interpretation prioritized the Group × Time interaction model.

Because treatment assignment was clinically determined, baseline-adjusted ANCOVAs were not interpreted as removing confounding or establishing causal effects. They were used only as supplementary sensitivity analyses to examine whether the observed pattern was similar after adjustment for the corresponding baseline outcome score. Propensity-score adjustment was considered but not implemented as a primary analytic strategy because several clinically relevant assignment factors—such as crisis instability, clinician judgment regarding need for structured stabilization, family engagement, treatment motivation, and therapist availability—were not systematically available in the retrospective dataset. Under these conditions, a propensity-score model based only on a limited set of measured baseline variables could have provided a misleading impression of causal adjustment. Caregiver preference was recorded only as a contextual assignment consideration rather than as a structured, analyzable variable, and related constructs such as family engagement, therapeutic alliance, and treatment motivation were not systematically measured. Consequently, the magnitude of caregiver-preference-related self-selection bias could not be defensibly modeled; qualitatively, its direction is ambiguous because greater engagement among families preferring one intervention could amplify apparent improvement, whereas preferences driven by greater perceived severity or crisis concern could bias estimates in the opposite direction.

Effect sizes for the ANOVA and ANCOVA models were reported as partial η^2^. Statistical significance was set at *p* < 0.05, two-tailed. Because multiple psychological outcomes were examined in this exploratory retrospective analysis, findings were interpreted cautiously, with emphasis on multiplicity-adjusted conclusions rather than isolated nominal *p*-values. Bonferroni and Benjamini–Hochberg false discovery rate procedures were applied as post hoc sensitivity checks for the seven Group × Time interaction tests and the seven supplementary ANCOVA group effects. Findings that did not remain significant after these multiplicity checks were interpreted as uncorrected exploratory signals rather than confirmatory evidence. To provide precision estimates, Supplementary Table S5 reports within-group mean changes, within-group paired Cohen’s d, and between-group change differences with 95% confidence intervals for all seven outcomes.

Linear mixed-effects models were considered in response to the concern regarding repeated-measures ANOVA. However, the present dataset included only two assessment time points and a complete-case analytic sample. Under this data structure, the fixed Group × Time contrast estimated in a basic linear mixed-effects model with a subject-level random intercept would be expected to yield conclusions substantively similar to those from the mixed-design ANOVA, whereas the principal limitations of the study arise from non-randomized clinical assignment, multiplicity, and unmeasured confounding rather than from the repeated-measures model structure itself. We therefore retained the mixed-design ANOVA as the primary descriptive longitudinal model and used baseline-adjusted ANCOVAs and expanded covariate-adjusted sensitivity analyses as supplementary checks. Missing data were handled using a complete-case approach for the relevant analyses.

Missing post-intervention data were not assumed to be missing completely at random, as the absence of post-treatment assessment may have reflected early discontinuation, incomplete assessment, disengagement, or escalation to higher-intensity care. Because post-intervention outcomes were unavailable for excluded cases and structured baseline data were inconsistent across exclusion stages, formal MCAR testing and imputation were not considered defensible. Complete-case findings should therefore be interpreted as applying to treatment-engaged completers rather than to all referred or treatment-initiating adolescents.

## 3. Results

### 3.1. Participant Characteristics

Of 491 school-linked referrals during the study period, 156 did not proceed to baseline assessment or the target intervention pathway; representative reasons included family or student refusal, preference for external care, logistical or academic barriers, and immediate referral to higher-intensity services before baseline assessment. Of the 335 cases assessed for analytic eligibility, 143 were excluded before analytic inclusion; representative reasons included no documented NSSI and/or suicidal ideation meeting study criteria, concurrent or alternative treatment, and acute psychiatric instability identified during eligibility assessment requiring higher-intensity care. Of the 192 adolescents who initiated SPT-SAFE or DBT-BI, 80 were excluded from the final analytic sample; representative reasons included insufficient intervention exposure, incomplete pre–post assessment data, and transfer or escalation to higher-intensity care during the treatment episode (n = 20). The final analytic sample therefore consisted of 112 adolescents who received at least six sessions and had complete pre–post assessment data (SPT-SAFE: n = 52; DBT-BI: n = 60). The complete participant flow is presented in Figure 1. Demographic characteristics of the final analytic sample are presented in Table 2.

Safety-related clinical escalation information is summarized in Supplementary Table S3. Among the final analytic sample, no documented emergency referral, psychiatric hospitalization, medically serious suicide attempt, or transfer/escalation to higher-intensity care occurred during the treatment episode. However, among the 192 adolescents who initiated SPT-SAFE or DBT-BI, 20 were transferred or escalated to higher-intensity care during the treatment episode and were therefore not retained in the final analytic sample.

Because the final analytic sample included only treatment-engaged completers with complete pre–post data, the absence of documented emergency referral, psychiatric hospitalization, or treatment-episode escalation within this analytic sample should not be interpreted as evidence that such adverse trajectories did not occur in the broader treatment-initiating population. These findings therefore apply only to treatment-engaged completers and may systematically underestimate adverse trajectories.

### 3.2. Baseline Comparability Between Groups

Baseline differences between the SPT-SAFE and DBT-BI groups were examined descriptively using independent-samples *t*-tests. No statistically significant differences were observed across the seven measured outcome variables (all *p* > 0.05).

However, consistent with recommendations for non-randomized studies, baseline comparability was interpreted primarily using standardized effect sizes rather than significance testing. Baseline effect sizes (Cohen’s d) ranged from −0.16 to 0.18, all within the small range, suggesting minimal differences between groups on measured outcome variables at intake.

These results indicate that the two groups were broadly comparable on observed baseline characteristics, although the possibility of differences in unmeasured factors cannot be excluded. Detailed results are presented in Table 3.

### 3.3. Mixed-Design ANOVA Results

#### 3.3.1. Primary Outcomes: Suicidal Ideation (SIQ-JR) and NSSI (FASM)

For suicidal ideation, a significant main effect of Time was observed, F(1, 110) = 40.945, *p* < 0.001, partial η^2^ = 0.271, indicating lower SIQ-JR scores at post-intervention across both groups. Neither the main effect of Group, F(1, 110) = 1.166, *p* = 0.283, partial η^2^ = 0.010, nor the Group × Time interaction, F(1, 110) = 0.855, *p* = 0.357, partial η^2^ = 0.008, was statistically significant.

For NSSI frequency, a significant main effect of Time was also observed, F(1, 110) = 24.374, *p* < 0.001, partial η^2^ = 0.181, indicating lower FASM scores at post-intervention across both groups. Neither the main effect of Group, F(1, 110) = 0.996, *p* = 0.320, partial η^2^ = 0.009, nor the Group × Time interaction, F(1, 110) = 0.038, *p* = 0.845, partial η^2^ < 0.001, was statistically significant.

#### 3.3.2. Secondary Outcomes

Among the secondary outcomes, significant main effects of Time were observed for depressive symptoms (CES-DC), F(1, 110) = 46.443, *p* < 0.001, partial η^2^ = 0.297; trait anxiety (STAI-T), F(1, 110) = 26.862, *p* < 0.001, partial η^2^ = 0.196; aggression (AQ), F(1, 110) = 9.011, *p* = 0.003, partial η^2^ = 0.076; self-concept (PHCSCS), F(1, 110) = 18.071, *p* < 0.001, partial η^2^ = 0.141; and impulsiveness (BIS), F(1, 110) = 15.007, *p* < 0.001, partial η^2^ = 0.120. These results indicate improvement over time across both groups, reflected by lower post-intervention scores for CES-DC, STAI-T, AQ, and BIS, and higher post-intervention scores for PHCSCS.

No significant main effect of Group was observed for any secondary outcome. Group × Time interactions were also non-significant for CES-DC, STAI-T, AQ, and PHCSCS. For BIS, however, a nominal Group × Time interaction was observed before multiplicity correction, F(1, 110) = 4.872, *p* = 0.029, partial η^2^ = 0.042. Descriptively, the DBT-BI group showed a larger mean reduction in BIS scores (Δ = 4.40) than the SPT-SAFE group (Δ = 1.12). In post hoc multiplicity sensitivity checks across the seven Group × Time interaction tests, this interaction did not remain statistically significant under either the Bonferroni-adjusted threshold (α = 0.007) or the Benjamini–Hochberg false discovery rate procedure. Therefore, the BIS interaction should be interpreted as an uncorrected exploratory finding.

**Figure 2:**
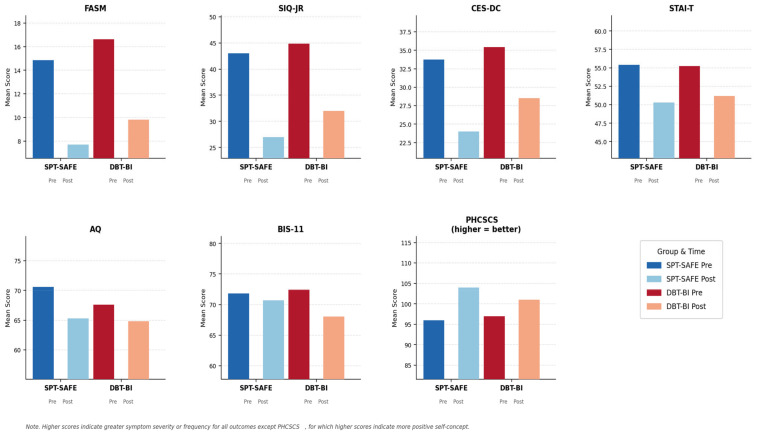
Pre- and post-intervention mean scores by group for all outcome measures.

**Figure 3:**
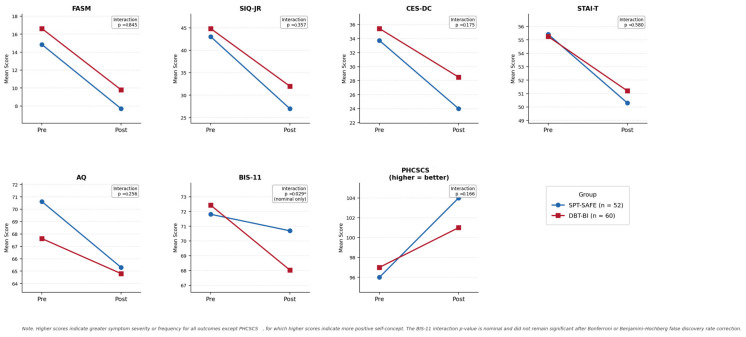
Group × Time interaction plots for all outcome measures. Note. * *p* < 0.05.

A summary of the mixed-design ANOVA results for all outcome measures is provided in Table 4. Pre–post changes for all outcome measures by group are illustrated in Figure 2, and the corresponding Group × Time interaction patterns are presented in Figure 3.

### 3.4. ANCOVA Results

To complement the mixed-design ANOVA findings, one-way ANCOVAs were conducted for post-intervention scores, with the corresponding baseline scores entered as covariates. Consistent with the Group × Time interaction observed in the mixed-design ANOVA, a baseline-adjusted between-group difference was observed for impulsiveness (BIS), F(1, 109) = 4.939, *p* = 0.028, partial η^2^ = 0.043, with lower adjusted post-intervention BIS scores in the DBT-BI group than in the SPT-SAFE group. However, this ANCOVA group effect did not remain statistically significant after Bonferroni or Benjamini–Hochberg false discovery rate correction across the seven supplementary ANCOVA models. No statistically significant between-group differences were observed for the remaining outcome measures after baseline adjustment (all *p* > 0.05).

**Figure 4:**
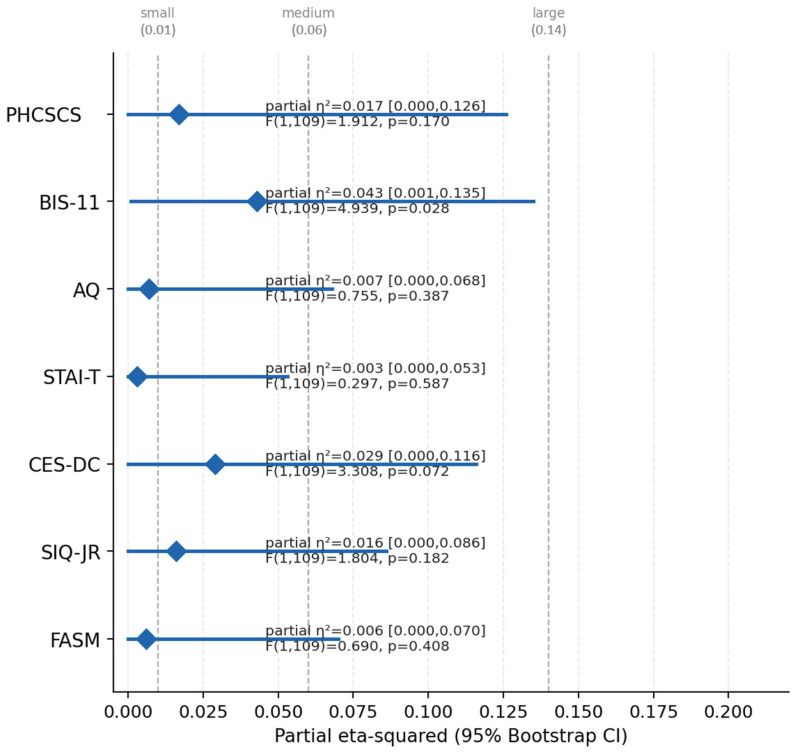
Forest plot of effect sizes (partial η^2^) from ANCOVA analyses controlling for baseline scores. Note. Partial η^2^ values indicate effect-size magnitude and do not indicate direction of change. PHCSCS = modified PHCSCS-based self-concept index; higher scores indicate a more positive self-concept. No between-group difference was statistically significant after Bonferroni or Benjamini–Hochberg false discovery rate correction.

These ANCOVA findings were considered supplementary and descriptive in the context of the present non-randomized observational design. Although baseline adjustment may reduce the influence of measured baseline differences, it cannot address unmeasured selection factors or confounding by indication. Therefore, the baseline-adjusted BIS result should be interpreted as an exploratory association rather than evidence of a causal or intervention-specific effect. An expanded covariate-adjusted sensitivity analysis (Supplementary Table S4) in which the BIS post-intervention score was regressed on Group with baseline BIS, age, sex, and baseline SIQ-JR, FASM, CES-DC, and AQ as covariates yielded a nominally significant group coefficient (B = −2.908, SE = 1.447, *p* = 0.047, R^2^ = 0.564); however, this finding does not eliminate the possibility of confounding by indication, as the clinical assignment variables most likely to produce confounding were not available in the retrospective dataset. Complete ANCOVA results are presented in Table 5 and Figure 4, and expanded covariate-adjusted sensitivity results are provided in Supplementary Table S4.

### 3.5. Summary of Outcome Patterns

Across the mixed-design ANOVA analyses, main effects of Time yielded medium-to-large effect sizes for suicidal ideation (partial η^2^ = 0.271), NSSI frequency (partial η^2^ = 0.181), depressive symptoms (partial η^2^ = 0.297), and trait anxiety (partial η^2^ = 0.196), indicating notable pre-to-post changes across both groups. The BIS Group × Time interaction was nominally significant before multiplicity correction (partial η^2^ = 0.042), but this effect was small and did not remain significant after Bonferroni or Benjamini–Hochberg false discovery rate correction. This pattern was broadly consistent with the supplementary baseline-adjusted ANCOVA results, but should be interpreted as an uncorrected exploratory signal given the retrospective design, multiple outcomes examined, and non-randomized clinical assignment process. Precision estimates for within-group changes and between-group change contrasts, including 95% confidence intervals, are provided in Supplementary Table S5.

## 4. Discussion

### 4.1. Overall Outcome Patterns

The present study examined outcome patterns associated with SPT-SAFE and DBT-BI in adolescents with suicidal ideation and non-suicidal self-injury in a school-based clinical setting. Across outcome domains, both interventions were associated with significant pre–post improvements, including reductions in suicidal ideation, NSSI frequency, and emotional distress. Between-intervention differences were generally limited.

An uncorrected Group × Time interaction was observed only for impulsiveness, with the DBT-BI group showing a descriptively larger reduction than the SPT-SAFE group. However, this effect was small in magnitude and did not remain statistically significant after Bonferroni or false discovery rate correction. Accordingly, the impulsivity finding should be interpreted as a preliminary, hypothesis-generating signal rather than evidence of differential efficacy.

Taken together, the findings indicate broadly comparable overall changes across the two intervention tracks, with limited evidence of systematic between-intervention differentiation. This pattern is consistent with a broader body of research showing that psychological interventions for adolescent self-harm and suicidality typically produce modest but clinically relevant improvements, often with limited differentiation between active treatment conditions (Fox et al., 2020; Ougrin et al., 2015; Wright-Hughes et al., 2025). In this context, the present results are best understood not as evidence of treatment superiority, differential effects across outcome domains, or equivalence between the two pathways, but as short-term improvements observed in both groups with limited evidence of between-group differences.

### 4.2. Shared Intervention Elements and Clinical Viability in a Higher-Severity Adolescent Sample

The broad within-group improvements observed across both intervention tracks are notable, particularly given the elevated clinical severity of the present middle- and high-school sample. Both SPT-SAFE and DBT-BI were associated with reductions in suicidal ideation, NSSI frequency, depressive symptoms, anxiety, and aggression, as well as improvements in self-concept, despite the relatively brief duration of the interventions and higher baseline symptom levels compared with the elementary-school sample examined in our prior retrospective study (Kwak & Ahn, 2026).

Both intervention tracks incorporated shared structural elements, including structured safety monitoring, therapeutic engagement, and caregiver–school coordination. These elements are widely recognized as central components of adolescent self-harm intervention and school-based mental health care (Fazel et al., 2014; Ougrin et al., 2015; Meza et al., 2023). These shared features may account for a substantial portion of the observed improvements across outcome domains, independent of modality-specific components.

The observed immediate pre–post improvements suggest short-term clinical viability of both intervention tracks within the school-based service context. However, because no follow-up assessments were available, the present study cannot determine whether these changes were maintained, whether post-treatment risk re-escalation occurred, or whether either intervention provided longer-term clinical utility. The absence of broad between-intervention superiority should therefore not be interpreted as evidence of clinical inefficacy; rather, it should be understood within the limits of immediate post-treatment outcomes and in light of prior evidence that multiple active interventions can produce comparable levels of meaningful change in adolescent self-harm populations (Fox et al., 2020; Wright-Hughes et al., 2025).

### 4.3. Limited Between-Intervention Differentiation and a Behavioral Regulation Signal

A nominal, uncorrected Group × Time interaction was observed for impulsiveness (BIS; partial η^2^ = 0.042), with the DBT-BI group showing a descriptively larger reduction than the SPT-SAFE group. However, this effect was small and did not remain statistically significant after Bonferroni correction (adjusted α = 0.007) or Benjamini–Hochberg false discovery rate correction across the seven interaction tests. Accordingly, this finding should not be interpreted as evidence of differential efficacy, domain-specific comparative efficacy, or a treatment-specific mechanism. Rather, it should be regarded as a small, uncorrected, exploratory signal for future hypothesis generation.

The four-outcome grouping used in this study was therefore an organizing structure for description only, not a framework confirmed by the present data. Theoretical links between DBT-informed skills and behavioral regulation are discussed only as background context, not as an explanation for the observed pattern (Linehan, 1993; Rathus & Miller, 2002). Confounding by indication also remains a plausible alternative explanation: adolescents perceived by clinicians as needing more structured behavioral stabilization may have been more likely to receive DBT-BI, and such clinical indications may also be related to short-term changes in impulsivity. Regression to the mean, measurement variability, and other unmeasured clinical differences may also have contributed to the observed BIS pattern. No other outcome showed a multiplicity-adjusted Group × Time interaction.

Taken together, the findings indicate broadly comparable improvement across most outcomes, with only a small, uncorrected exploratory signal emerging for impulsiveness.

This pattern may inform future research, but it does not establish differential treatment effects or domain-specific comparative efficacy.

### 4.4. Cautions and Directions for Future School-Based Intervention Research

The present findings do not provide a sufficient basis for using a domain-sensitive perspective as a framework for intervention planning. Because adolescents with suicidal ideation and NSSI present with heterogeneous clinical profiles, describing change across multiple outcomes may, at most, serve as a starting point for future hypothesis generation rather than as a basis for prescriptive or age-based treatment matching. Cross-cohort observations relative to the prior elementary-school study (Kwak & Ahn, 2026) are noted only as an exploratory context and are not interpreted as evidence of age-specific treatment effects or confirmed developmental trajectories.

### 4.5. Strengths and Limitations

The present study has several notable strengths. First, it was conducted within a naturalistic school-based clinical setting, which enhances the ecological validity of the findings and their relevance to real-world intervention planning. Unlike efficacy trials conducted under highly controlled conditions, the present findings reflect outcome patterns observed under the operational constraints typical of school-based mental health services, including limited session duration, ongoing safety monitoring, and coordination with caregivers and school personnel. Second, the use of multiple validated outcome measures across distinct functional domains—including suicidal ideation, NSSI frequency, behavioral regulation, emotional distress, and self-concept—allowed for a domain-sensitive examination of intervention-associated change beyond global symptom indices. Third, the present study extends a prior retrospective observational study conducted within the same service system (Kwak & Ahn, 2026), providing a clinically grounded basis for examining whether outcome patterns may vary across developmental and severity contexts. Fourth, both intervention tracks were implemented within a structured clinical decision-making framework with documented safety protocols, enhancing the internal consistency of the service delivery context.

The most important limitation is the retrospective, non-randomized design and the resulting risk of confounding by indication. Intervention assignment was embedded in routine clinical decision-making rather than random allocation. Although measured baseline differences were described using SMDs and supplementary baseline-adjusted analyses were conducted, these approaches cannot establish exchangeability or rule out unmeasured selection factors. In particular, adolescents assigned to DBT-BI may have been perceived as having greater behavioral dysregulation, higher observed or clinician-perceived impulsivity, greater crisis instability, or a stronger need for structured behavioral stabilization, and these same factors may have contributed to the observed reduction in impulsivity. Confounding by indication therefore remains the most plausible specific alternative explanation for the nominal BIS finding and should be considered before any causal or intervention-specific interpretation is drawn. Accordingly, between-group comparisons should be interpreted as exploratory and not as evidence of comparative efficacy. Because the final analytic sample was a treatment-engaged completer sample rather than a representative sample of all school-linked referrals or treatment-initiating adolescents, selection bias and complete-case bias cannot be ruled out. Adolescents who discontinued treatment, had incomplete post-intervention assessments, or were escalated to higher-intensity care were not retained in the analytic sample. Therefore, the present findings may systematically underestimate adverse trajectories and should not be interpreted as sufficient evidence of safety in the broader treatment-initiating population. Second, only immediate post-treatment outcomes were available; therefore, conclusions about durability, post-treatment risk re-escalation, and longer-term clinical utility cannot be drawn. In this population, immediate post-intervention scores alone cannot determine whether clinical gains were maintained or whether self-harm or suicide-related risk re-escalated after treatment ended. Future studies should include structured follow-up assessments to evaluate the durability of school-based intervention effects. Third, the study was conducted within a single provincial, school-linked service system in South Korea, which limits generalizability to other cultural, clinical, and institutional contexts. Although the center functioned as a regional referral and intervention hub receiving students from urban, suburban, and rural schools across Chungcheongnam-do, transferability to other settings may depend on the presence of comparable school referral pathways, brief intervention structures, suicide-risk monitoring procedures, caregiver–school coordination, therapist training, and supervision systems. In addition, SPT-SAFE is grounded in established sandplay therapy principles but was structured for use within a school-linked suicide and NSSI intervention context; therefore, the transportability of this structured school-linked application of sandplay therapy should be examined through independent replication in other sites and cultural contexts, preferably using prospective designs. Fourth, therapeutic processes and mechanisms of change were not directly assessed, leaving open the question of whether the observed domain-specific patterns reflect the theoretically proposed components of each intervention or nonspecific factors. Fifth, although Bonferroni and Benjamini–Hochberg false discovery rate procedures were applied as post hoc sensitivity checks, the study was not designed around a prespecified multiplicity-controlled primary outcome. Therefore, isolated nominal findings, particularly the BIS interaction, remain vulnerable to Type I error and should be interpreted cautiously.

The study may also have been underpowered to detect clinically meaningful Group × Time interaction effects across multiple outcomes. Therefore, non-significant Group × Time interactions should not be interpreted as evidence of equivalence or comparable efficacy between SPT-SAFE and DBT-BI. Such findings indicate only that robust differential effects were not detected in this retrospective sample; clinically meaningful differential effects may have been missed, reflecting the risk of Type II error. This concern should be considered alongside the risk of Type I error for the nominal BIS finding, which did not survive multiplicity correction. Future prospective studies should include a priori power calculations for interaction effects and recruit larger samples capable of evaluating differential treatment effects with adequate precision.

A further limitation concerns treatment fidelity and adherence assessment. Although manual-derived fidelity/adherence checklists were developed for both SPT-SAFE and DBT-BI and used to guide supervision and monitoring, fidelity assessment was limited by the retrospective nature of the study. The archival dataset did not include full-session recordings, validated external fidelity instruments, fidelity ratings for every session, or systematically compiled quantitative fidelity scores. Therefore, we cannot fully rule out the possibility that observed outcome patterns were influenced by therapist-related differences, nonspecific therapeutic factors, or shared safety-management procedures rather than intervention-specific components. Future prospective studies should incorporate fully manualized intervention procedures, documented therapist training, systematic supervision logs, independent fidelity ratings, and session-level adherence monitoring across a larger proportion of sessions. Another limitation concerns the modified self-concept measure. Although no newly written items were introduced and internal consistency was good in the present sample, the 30-item, 6-point Likert format has not been independently validated; therefore, self-concept findings should be interpreted only as exploratory secondary outcome data and not as equivalent to PHCSCS-2 norm-based scores or cutoffs.

Taken together, these considerations indicate that the present findings are best understood as preliminary and hypothesis-generating. Future research should employ prospective or randomized designs with adequate statistical power, incorporate follow-up assessments, and include process-level measures to clarify how developmental stage, clinical severity, and functional domains interact in shaping intervention response in adolescents with suicidal ideation and non-suicidal self-injury.

## 5. Conclusions

In conclusion, this retrospective school-based study provides preliminary descriptive evidence regarding short-term symptom change among middle- and high-school adolescents receiving one of two school-based intervention pathways, SPT-SAFE or DBT-BI. Both intervention groups showed short-term improvements across several outcomes, but the study did not provide robust multiplicity-adjusted evidence for differential treatment effects between the two pathways. The nominal impulsivity finding should therefore be interpreted only as a small, uncorrected, exploratory observation for future hypothesis generation; it does not establish a mechanism of change, a modality-specific treatment effect, or domain-specific comparative efficacy and should not be used to support domain-sensitive intervention planning. The contribution of this study is descriptive and hypothesis-generating, intended to inform future prospective, adequately powered, and developmentally stratified research.

## Figures and Tables

**Table 1:** Baseline demographic and clinical characteristics at intake relevant to clinical assignment.

Domain	Variable	SPT-SAFE (n = 52)	DBT-BI (n = 60)	Absolute SMD
Demographics	Age (years)	14.62 ± 1.85	15.05 ± 1.89	0.23
	Male sex (%)	14 (26.9)	18 (30.0)	0.07
Clinical severity	SIQ-JR (baseline)	43.04 ± 21.97	44.83 ± 21.46	0.08
	CES-DC (baseline)	33.73 ± 10.65	35.43 ± 10.78	0.16
	STAI-T (baseline)	55.42 ± 7.92	55.25 ± 8.52	0.02
NSSI frequency	FASM (baseline)	14.85 ± 14.06	16.63 ± 14.45	0.13
Behavioral dysregulation	AQ (baseline)	70.62 ± 17.56	67.63 ± 16.40	0.18
	BIS (baseline)	71.81 ± 10.63	72.43 ± 10.54	0.06

Note. Data are presented as mean ± standard deviation or percentage, as appropriate. Standardized mean differences (SMDs) are reported as absolute values; smaller values indicate better baseline balance between groups. SMDs are presented to describe measured baseline differences and should not be interpreted as evidence that the clinically assigned groups were exchangeable. Unmeasured or partially documented selection factors—including behavioral dysregulation severity, crisis instability, and clinician judgment regarding need for structured stabilization—may have influenced both intervention assignment and subsequent outcomes.

**Table 2:** Participant demographic characteristics.

Characteristic	SPT-SAFE (n = 52)	DBT-BI (n = 60)	Total (n = 112)
Age M (SD)	14.62 (1.85)	15.05 (1.89)	14.85 (1.88)
Male n (%)	14 (26.9%)	18 (30.0%)	32 (28.6%)
Female n (%)	38 (73.1%)	42 (70.0%)	80 (71.4%)

**Table 3:** Baseline comparability testing results.

Variable	*t*	df	*p*	Cohen’s d	Baseline Difference
SIQJR	−0.437	110	0.663	−0.083	Not significant
FASM	−0.661	110	0.510	−0.125	Not significant
CESDC	−0.838	110	0.404	−0.159	Not significant
STAIT	0.111	110	0.912	0.021	Not significant
AQ	0.929	110	0.355	0.176	Not significant
BIS	−0.312	110	0.756	−0.059	Not significant
PHCSCS	−0.208	110	0.836	−0.039	Not significant

Note. Independent-samples *t*-tests were conducted to assess baseline comparability between the SPT-SAFE and DBT-BI groups. All Levene’s tests for equality of variances were non-significant (all *p* > 0.40).

**Table 4:** Mixed ANOVA results for all outcome measures.

Variable	Group Effect	Time Effect	Interaction Effect
SIQ-JR	F = 1.166, *p* = 0.283, partial η^2^ = 0.010	F = 40.945, *p* < 0.001, partial η^2^ = 0.271	F = 0.855, *p* = 0.357, partial η^2^ = 0.008
FASM	F = 0.996, *p* = 0.320, partial η^2^ = 0.009	F = 24.374, *p* < 0.001, partial η^2^ = 0.181	F = 0.038, *p* = 0.845, partial η^2^ < 0.001
CES-DC	F = 2.952, *p* = 0.089, partial η^2^ = 0.026	F = 46.443, *p* < 0.001, partial η^2^ = 0.297	F = 1.867, *p* = 0.175, partial η^2^ = 0.017
STAI-T	F = 0.041, *p* = 0.840, partial η^2^ < 0.001	F = 26.862, *p* < 0.001, partial η^2^ = 0.196	F = 0.308, *p* = 0.580, partial η^2^ = 0.003
AQ	F = 0.197, *p* = 0.658, partial η^2^ = 0.002	F = 9.011, *p* = 0.003, partial η^2^ = 0.076	F = 1.306, *p* = 0.256, partial η^2^ = 0.012
BIS	F = 0.290, *p* = 0.591, partial η^2^ = 0.003	F = 15.007, *p* < 0.001, partial η^2^ = 0.120	F = 4.872, *p* = 0.029, partial η^2^ = 0.042
PHCSCS	F = 0.194, *p* = 0.661, partial η^2^ = 0.002	F = 18.071, *p* < 0.001, partial η^2^ = 0.141	F = 1.940, *p* = 0.166, partial η^2^ = 0.017

Note. SPT-SAFE n = 52; DBT-BI n = 60; Total N = 112. Main effects of Time were observed across all seven outcome measures (all *p* < 0.01). The BIS Group × Time interaction was nominally significant before multiplicity correction (*p* = 0.029) but did not remain significant after Bonferroni correction (adjusted α = 0.007) or Benjamini–Hochberg false discovery rate correction across the seven interaction tests. Accordingly, this finding should be interpreted as an uncorrected exploratory signal rather than as confirmatory evidence of a differential treatment effect.

**Table 5:** ANCOVA Results controlling for baseline scores.

Variable	Group F	*p*	Partial η^2^	Adjusted Mean SPT	Adjusted Mean DBT	Multiplicity-Adjusted Interpretation
SIQ-JR	1.804	0.182	0.016	27.59	32.56	Not significant
FASM	0.690	0.408	0.006	7.85	9.64	Not significant
CES-DC	3.308	0.072	0.029	24.70	28.61	Not significant
STAI-T	0.297	0.587	0.003	50.30	51.22	Not significant
AQ	0.755	0.387	0.007	63.47	65.83	Not significant
BIS	4.939	0.028	0.043	70.94	67.82	Nominal only; not significant after Bonferroni or FDR correction
PHCSCS	1.912	0.170	0.017	104.67	100.77	Not significant

Note. After adjusting for baseline scores, a nominally significant between-group difference was observed for BIS only (*p* = 0.028); however, this effect did not remain statistically significant after Bonferroni or Benjamini–Hochberg false discovery rate correction across the seven supplementary ANCOVA models, and should therefore be interpreted as an uncorrected exploratory association rather than as evidence of a differential treatment effect. Values represent estimated marginal means evaluated at the grand mean of each baseline covariate.

## Data Availability

The data presented in this study are available on reasonable request from the corresponding author. The data are not publicly available due to privacy and ethical restrictions, as they were derived from de-identified archival clinical records of minors receiving school-linked mental health services for suicidal ideation and non-suicidal self-injury. Requests for data access will be considered in accordance with the Institutional Review Board approval, institutional regulations, and applicable privacy protections.

## Supplementary Materials

The following supporting information can be downloaded at: https://www.mdpi.com/article/10.3390/bs16060916/s1, Table S1. SPT-SAFE Treatment Fidelity Checklist; Table S2. DBT-BI Treatment Fidelity Checklist; Table S3. Safety Events and Higher-Intensity Care Escalation During the Treatment Episode; Table S4. Expanded Covariate-Adjusted Sensitivity Analysis for BIS Post-Intervention Score; Table S5. Precision Estimates for Within-Group Pre–Post Changes and Between-Group Change Contrasts.

## Abbreviations

The following abbreviations are used in this manuscript:

SPT-SAFE	Sandplay Therapy with Suicidal ideation and Self-injury Focused Engagement
DBT-BI	Dialectical Behavior Therapy-informed Brief Intervention
NSSI	Non-Suicidal Self-Injury
SIQ-JR	Suicidal Ideation Questionnaire–Junior
CES-DC	Center for Epidemiologic Studies Depression Scale for Children
STAI-T	State–Trait Anxiety Inventory for Children—Trait
PHCSCS	Piers–Harris Children’s Self-Concept Scale
FASM	Functional Assessment of Self-Mutilation
