# Supplementary material for: Outcome Patterns of SPT-SAFE and DBT-BI in Adolescents with Suicidal Ideation and Non-Suicidal Self-Injury: A Retrospective School-Based Study
Source: Behav Sci (Basel). 2026 Jun 3;16(6):916. doi: 10.3390/bs16060916 (PMC13295367; doi:10.3390/bs16060916)
Supplement: Supplementary file 1 [file behavsci-16-00916-s001.zip › behavsci-4312350-supplementary.pdf]

Supplementary Materials

Table S1. SPT-SAFE Treatment Fidelity Checklist (Manual-Derived)

No.	Fidelity Domain	Fidelity Item (Assessment Criterion)	Rating Method
1	Therapeutic Setting	The therapist maintained a non-directive stance and provided a free and protected therapeutic space that allowed autonomous symbolic expression by the client.	0 = Not adhered<br>1 = Partially adhered<br>2 = Fully adhered
2	Core Sandplay Components	Standard sandtray materials (sandtray, miniatures, and symbolic objects) were appropriately used in accordance with the SPT-SAFE manual.	0 / 1 / 2
3	Proscribed Elements	The therapist refrained from imposing interpretations or attributing explicit meanings to the client's symbolic expressions.	0 / 1 / 2
4	Risk Monitoring	Assessment of suicidal ideation and/or non-suicidal self-injury (NSSI) risk was conducted during the session in accordance with the SPT-SAFE risk-focused framework.	0 / 1 / 2
5	Safety Planning Integration	When indicated, individualized safety planning strategies were appropriately addressed or reinforced within the therapeutic process.	0 / 1 / 2
6	Therapist Stance	The therapist consistently demonstrated empathic witnessing, a non-judgmental attitude, and emotional containment.	0 / 1 / 2
7	Clinical Documentation	Session content, therapeutic interventions, and risk-related observations were accurately and comprehensively documented in structured clinical records.	0 / 1 / 2

Note. This manual-derived fidelity checklist was developed from the core components of the SPT-SAFE intervention. Items were rated session-by-session on a 3-point scale (0 = not adhered, 1 = partially adhered, 2 = fully adhered), and session-level scores were calculated as percentages. Approximately 20% of sessions were independently reviewed. Because this retrospective archival study did not include systematically compiled fidelity ratings, aggregate adherence statistics cannot be reported. The checklist is therefore provided to document the fidelity framework, and the absence of quantitative fidelity data is acknowledged as a study limitation.

Table S2. DBT-BI Treatment Fidelity Checklist (Manual-Derived)

No.	Fidelity Domain	Fidelity Item (Assessment Criterion)	Rating Method
1	Session Structure	The therapist followed the prescribed clinical sequence, beginning with brief mindfulness-based stabilization before proceeding to behavioral monitoring and skills work.	0 = Not adhered<br>1 = Partially adhered<br>2 = Fully adhered
2	Behavioral Monitoring	Suicidal ideation and NSSI behaviors were systematically reviewed using a diary card, and priority risk targets were identified and hierarchized in accordance with the DBT-BI protocol.	0 / 1 / 2
3	Chain Analysis	A DBT-based chain analysis was conducted to identify antecedents, vulnerability factors, and consequences of target risk behaviors, with findings used to guide subsequent skill selection.	0 / 1 / 2
4	Skills Practice	Selected DBT skills (e.g., emotion regulation, distress tolerance) were collaboratively practiced to address identified target behaviors, with skill selection individualized based on ongoing behavioral assessment.	0 / 1 / 2
5	Therapeutic Stance	The therapist consistently demonstrated a dialectical balance of validation and change strategies, maintaining a non-judgmental, collaborative, and behaviorally focused therapeutic posture.	0 / 1 / 2
6	Safety Planning Integration	When clinically indicated, individualized safety plans were reviewed, updated, or reinforced, and predefined crisis response procedures were activated if imminent risk was identified.	0 / 1 / 2
7	Clinical Documentation	Session content, diary card data, chain analysis findings, skills practiced, and risk-related clinical observations were accurately and comprehensively documented in structured clinical records.	0 / 1 / 2

Note. This manual-derived fidelity checklist was developed from the core components of the DBT-BI intervention protocol. Items were rated session-by-session on a 3-point scale (0 = not adhered, 1 = partially adhered, 2 = fully adhered), and session-level scores were calculated as percentages. Approximately 20% of sessions were independently reviewed. Because this retrospective archival study did not include systematically compiled fidelity ratings, aggregate adherence statistics cannot be reported. The checklist is therefore provided to document the fidelity framework, and the absence of quantitative fidelity data is acknowledged as a study limitation.

Table S3. Safety Events and Higher-Intensity Care Escalation During the Treatment Episode

Safety Indicator	Total
**Treatment Initiators**	
**Treatment initiators**	**N = 192**
SPT-SAFE initiated	n = 93
DBT-BI initiated	n = 99
**Excluded From Final Analytic Sample**	
**Excluded from final analytic sample**	**n = 80**
SPT-SAFE excluded	n = 41
DBT-BI excluded	n = 39
Transfer/escalation to higher-intensity care during the treatment episode	n = 20
**Final Analytic Sample**	
**Final analytic sample**	**N = 112**
SPT-SAFE final analytic sample	n = 52
DBT-BI final analytic sample	n = 60
**Safety Events Among Final Analytic Sample**	
Documented emergency referral	n = 0
Documented psychiatric hospitalization	n = 0
Documented medically serious suicide attempt	n = 0
Documented transfer/escalation to higher-intensity care	n = 0

Note. SPT-SAFE = Sandplay Therapy with Suicidal Ideation and Self-Injury–Focused Engagement; DBT-BI =

Dialectical Behavior Therapy–informed Brief Intervention.

Safety-related events are reported separately for treatment initiators and for the final analytic sample. Escalation to higher-intensity care refers to transfer or referral to emergency, psychiatric, or other higher-intensity clinical services during the treatment episode. Adolescents who were transferred or escalated to higher-intensity care were not retained in the final complete-case analytic sample.

Table S4. Expanded Covariate-Adjusted Sensitivity Analysis for BIS Post-Intervention Score

Model: Post-BIS = Group + baseline BIS + Age + Sex + baseline SIQ-JR + baseline FASM + baseline CES-DC + baseline AQ

$R^2 = .564$, Adjusted $R^2 = .530$, N = 112

Variable	B	SE	t	p	95% CI LL	95% CI UL	Sig.
Intercept	22.804	9.195	2.480	.015	4.569	41.040	—
Group (DBT-BI)	−2.908	1.447	−2.010	.047	−5.779	−0.038	*
BIS (baseline)	0.770	0.075	10.222	<.001	0.621	0.919	***
Age	−0.064	0.401	−0.161	.873	−0.860	0.731	
Sex	−1.027	1.735	−0.592	.555	−4.467	2.413	
SIQ-JR (baseline)	−0.017	0.042	−0.411	.682	−0.101	0.066	
FASM (baseline)	−0.006	0.061	−0.093	.926	−0.127	0.115	
CES-DC (baseline)	−0.114	0.100	−1.147	.254	−0.312	0.083	
AQ (baseline)	0.000	0.054	0.001	.999	−0.107	0.107	

Note. The dependent variable was post-intervention BIS score. Group was coded as 0 = SPT-SAFE and 1 = DBT-BI; therefore, a negative group coefficient indicates a lower adjusted post-intervention BIS score in the DBT-BI group relative to the SPT-SAFE group. This analysis was conducted as an exploratory covariate-adjusted sensitivity analysis only. Because intervention assignment was clinically determined and key assignment factors were not systematically available as structured variables, this model should not be interpreted as removing confounding or establishing a causal treatment effect.

* $p < .05$. ** $p < .01$. *** $p < .001$.

Table S5. Precision Estimates for Within-Group Pre–Post Changes and Between-Group Change Contrasts

Table S5a. Within-Group Mean Changes and Cohen's d

Outcome	Group	n	Within-Group Mean Change				Within-Group Cohen's d (paired)		
			M change	SD	95% CI LL	95% CI UL	d	95% CI LL	95% CI UL
SIQ-JR	SPT-SAFE	52	-15.885	22.683	-22.050	-9.719	-0.700	-1.004	-0.397
	DBT-BI	60	-11.900	22.791	-17.667	-6.133	-0.522	-0.792	-0.252
FASM	SPT-SAFE	52	-7.288	14.268	-11.167	-3.410	-0.511	-0.800	-0.222
	DBT-BI	60	-6.733	15.580	-10.676	-2.791	-0.432	-0.697	-0.168
CES-DC	SPT-SAFE	52	-9.538	11.023	-12.535	-6.542	-0.865	-1.184	-0.547
	DBT-BI	60	-6.383	13.111	-9.701	-3.066	-0.487	-0.754	-0.219
STAI-T	SPT-SAFE	52	-5.058	6.907	-6.935	-3.180	-0.732	-1.038	-0.426
	DBT-BI	60	-4.083	10.894	-6.840	-1.327	-0.375	-0.637	-0.113
AQ	SPT-SAFE	52	-6.038	13.761	-9.779	-2.298	-0.439	-0.723	-0.154
	DBT-BI	60	-2.767	16.185	-6.862	1.329	-0.171	-0.426	0.084
BIS	SPT-SAFE	52	-1.115	7.522	-3.160	0.929	-0.148	-0.422	0.125
	DBT-BI	60	-4.400	8.131	-6.457	-2.343	-0.541	-0.812	-0.270
PHCSCS	SPT-SAFE	52	8.365	13.008	4.830	11.901	0.643	0.344	0.942
	DBT-BI	60	4.300	17.208	-0.054	8.654	0.250	-0.007	0.507

Table S5b. Between-Group Change Differences

Outcome	Between-Group Change Difference (DBT-BI minus SPT-SAFE)		
	Difference	95% CI LL	95% CI UL
SIQ-JR	3.985	-4.457	12.427
FASM	0.555	-4.975	6.085
CES-DC	3.155	-1.315	7.625
STAI-T	0.974	-2.361	4.310
AQ	3.272	-2.275	8.818
BIS	**-3.285**	**-6.185**	**-0.384**
PHCSCS	-4.065	-9.674	1.544

Note. Change scores were calculated as post-intervention minus pre-intervention scores; negative values indicate decreases and positive values indicate increases. For PHCSCS, positive change indicates improvement (higher scores reflect more positive self-concept). Cohen's d (paired) was calculated as M_change / SD_change. 95% CIs for within-group mean changes and between-group change differences were calculated as $M \pm 1.96 \times SE$. 95% CIs for Cohen's d were estimated using the standard error approximation $SE_d = \sqrt{(1/n + d^2/2n)}$. Between-group change differences were calculated as DBT-BI change minus SPT-SAFE change; a negative value indicates a larger decrease (or smaller increase) in the DBT-BI group. The BIS row (highlighted) corresponds to the only nominally significant Group × Time interaction ($p = .029$, uncorrected); this interaction did not remain significant after Bonferroni or Benjamini–Hochberg false discovery rate correction across the seven interaction tests and should be interpreted as an uncorrected exploratory signal. Confidence intervals are provided to make the uncertainty of estimates transparent and should not be interpreted as multiplicity-adjusted or causal estimates. SE_d = standard error of Cohen's d. SPT-SAFE = Sandplay Therapy with Suicidal Ideation and Self-Injury–Focused Engagement; DBT-BI = Dialectical Behavior Therapy–informed Brief Intervention; CI = confidence interval; LL = lower limit; UL = upper limit.
